# Insecticidal Activity of *Allium sativum* Essential Oil-Based Nanoemulsion against *Spodoptera littoralis*

**DOI:** 10.3390/insects15070476

**Published:** 2024-06-26

**Authors:** Gaetano Giuliano, Orlando Campolo, Giuseppe Forte, Alberto Urbaneja, Meritxell Pérez-Hedo, Ilaria Latella, Vincenzo Palmeri, Giulia Giunti

**Affiliations:** 1 Department of Agriculture, University of Reggio Calabria, Feo di Vito, 89122 Reggio Calabria, Italy; gaetano.giuliano@unirc.it (G.G.); orlando.campolo@unirc.it (O.C.); ilaria.latella@unirc.it (I.L.); vpalmeri@unirc.it (V.P.); 2 Agrigeos s.r.l., Via Giordano Bruno 136, 95131 Catania, Italy; forte@agrigeos.com; 3 Instituto Valenciano de Investigaciones Agrarias (IVIA), Centro de Protección Vegetal y Biotecnología, Unidad de Entomología, 46113 Moncada, Spainperez_merhed@gva.es (M.P.-H.); 4 Department of Pharmacy, University of Salerno, Via Giovanni Paolo II 132, 84084 Fisciano, Italy

**Keywords:** antifeedant, biopesticides, cotton leafworm, garlic, phytotoxicity

## Abstract

**Simple Summary:**

*Spodoptera littoralis*, the Egyptian or African cotton leafworm, is a major agricultural pest in Africa, Mediterranean Europe, and the Middle East, affecting crops like cotton, soybeans, and tomatoes. This pest damages various plant parts, leading to significant product losses. Control strategies primarily use synthetic insecticides, which pose problems such as resistance, environmental harm, and negative effects on non-target organisms. This study explores a garlic essential oil-based nanoemulsion as an alternative control tool. Garlic-EO nanoemulsion effectively killed larvae and reduced feeding activity in laboratory trials. These findings highlight the potential of botanical insecticides for sustainable pest management.

**Abstract:**

*Spodoptera littoralis*, commonly known as the Egyptian or African cotton leafworm, is a significant agricultural threat. It is widely distributed in Africa, Mediterranean Europe, and Middle Eastern countries. This polyphagous pest infests numerous crop plants across 44 families, including cotton, soybeans, alfalfa, sweet potato, pepper, eggplant, tomato, maize, lettuce, strawberry, wheat, and hibiscus. The damage caused by *S. littoralis* on different plant organs, such as young leaves, shoots, stalks, bolls, buds, and fruits, often determines substantial product losses. Current control strategies predominantly rely on synthetic insecticides, which, despite their efficacy, have notable drawbacks, including insecticide resistance, environmental contamination, consumer concerns, and adverse effects on non-target organisms and beneficial insects. In response to these challenges, in this study, we developed and evaluated a garlic EO-based nanoemulsion with a high EO concentration (15%) and low surfactant content to mitigate the possible negative impact on plants and to enhance efficacy against *S. littoralis* larvae. Laboratory bioassays demonstrated promising larvicidal activity and reduced larval feeding, although some phytotoxicity symptoms were observed. This study underscores the potential of botanical insecticides as sustainable alternatives to synthetic chemicals, emphasizing the importance of balancing efficacy with environmental and ecological considerations in pest management strategies.

## 1. Introduction

*Spodoptera littoralis* Boisduval (Lepidoptera: Noctuidae), known as the Egyptian or African cotton leafworm, is widely distributed in Africa, Mediterranean regions of Europe and Middle Eastern countries [1]. This moth is a polyphagous pest of various ornamental plants and horticultural crops, infesting dozens of cultivated plants belonging to 44 families, including cotton, soybeans, alfalfa, sweet potato, pepper, eggplant, tomato, maize, lettuce, and strawberry, as well as wheat and hibiscus [2]. Damage is caused by larvae trophic activity, which feed on young leaves, shoots, stalks, bolls, buds, and fruits [3]. Its infestation can also produce the subsequent development of pathogens on damaged plant tissues and can lead to significant product losses [4]. Nowadays, the most common control strategies used against this pest rely on applying synthetic insecticides. Different groups of insecticides, such as organophosphates, pyrethroids, carbamates and insect growth regulators (IGRs), as well as several newer insecticides, including indoxacarb and chlorantraniliprole, are commonly used to control *S. littoralis* [5]. However, these approaches present some drawbacks, as those synthetic molecules can contribute to the emergence of insecticide resistance, environmental pollution, consumer rejection, and adverse effects on non-target organisms and beneficial insects [6,7]. Resistance has already been proved for most of the above-cited synthetic insecticides, mainly due to the development of acetylcholinesterase enzyme (AChE) insensitivity or metabolic detoxification enzymes [8]. The eco-toxicological, environmental, and social implications arising from the extensive and sometimes indiscriminate use of synthetic insecticides in agriculture have encouraged researchers to find more sustainable alternatives to conventional pesticides. Bioinsecticides, including *Bacillus thuringiensis*, spinosad, and spinetoram, are commonly applied in fields to prevent *S. littoralis* damage, although resistance toward those bioactive substances has already been recorded in wild pest populations [8]. Among these alternatives, botanical extracts, particularly essential oils (EOs), have emerged as a promising option for pest control. Their potential lies in their wide availability and relative affordability, as well as in their intrinsic insecticidal properties, which derive from the defensive role of these molecules in plant physiology [9].

One of the primary advantages of EOs is their acknowledged low toxicity to humans and non-target organisms. Unlike conventional synthetic insecticides, which often pose health and environmental risks, these blends of compounds are generally considered safe when used appropriately. They offer a sustainable and eco-friendly approach to pest management, aligning with integrated pest management (IPM) principles and organic farming practices [10]. Furthermore, EOs can exhibit broad-spectrum activity, targeting multiple pests across different insect orders. Their complex chemical compositions often include various bioactive compounds that act synergistically, reducing the possibility of insects establishing resistance. These characteristics make EOs valuable tools for controlling pest populations and reducing reliance on synthetic chemicals [11]. Moreover, EOs can offer additional benefits beyond insecticidal activity. Some of them possess repellent properties, deterring insects from treated areas, while others may exhibit antimicrobial or antifungal effects, reducing the risk of secondary infections by plant pathogens [12]. However, it is crucial to acknowledge the limitations associated with EO-based insecticides. One key drawback is their relatively short persistence since EO volatile compounds evaporate or degrade rapidly, limiting their residual activity. Frequent reapplication may be necessary, which can be labor-intensive and increase production costs [13]. In this context, the proper development and evaluation of the insecticidal efficacy of plant molecules or extracts could play an important role in developing innovative techniques and tools for controlling this key pest [14].

The main objective of this study was to investigate the insecticidal activity of garlic EO against *S. littoralis* larvae. Garlic, *Allium sativum* L. (Amaryllidaceae), is a globally cultivated crop primarily used in the food and pharmaceutical industries. In the literature, the efficacy of garlic EO has been investigated against several pests. Indeed, *A. sativum* EO possesses a recognized nematocidal activity [15], and its major constituents (organosulfur compounds) could act as acaricides against eriophyid mites [16,17] and as insecticides against stored product and crop insect pests [18,19]. The large availability, coupled with the promising bioactivity of either garlic aqueous extracts or EOs, made this plant a key candidate for developing innovative eco-friendly insecticides [20,21]. In this context, we have developed a garlic EO-based nanoemulsion characterized by a high amount of active ingredient (i.e., 15% of EO) and a low content of surfactant (Tween 80). The formulation underwent physical characterization using a dynamic light scattering (DLS) apparatus. The study aimed to evaluate the mortality rates induced by the formulated garlic EO, and to estimate the lethal concentrations (LCs) causing 50% and 90% mortality in the tested insect population, its phagodeterrent effects against *S. littoralis* larvae, and the possible phytotoxic impact on target plants.

## 2. Materials and Methods

### 2.1. Insects and Plant Rearing

The laboratory strain of *S. littoralis* used in this study was sourced from the agricultural research center Agrigeos s.r.l. (Acireale, Italy). The larvae were reared under laboratory conditions on an artificial diet (41.4 g/L wheat germ, 59.2 g/L brewer’s yeast, 165 g/L cornmeal, 5.9 g/L ascorbic acid, 1.53 g/L benzoic acid, 1.8 g/lmethyl 4-hydroxybenzoate and 29.6 g/L agar), as suggested by Caccia et al. [22], while the adults were provided with a sugar solution (1:1 *w*:*w*). All experiments used bell pepper plants (*Capsicum annuum* L., cv Makko F1). The plants used in the study were grown from untreated seeds and planted in pots filled with organic soil. The plants were regularly watered throughout the growth period and received the required nutrients. Both the insects and the plants used in the experiments were maintained under controlled constant conditions, with a temperature of 24 ± 2 °C, relative humidity (RH) of 50% ± 10, and a photoperiod of 14:10 h (L:D).

### 2.2. Development and Characterization of Garlic EO-Based Nano-Formulation

To assess the effectiveness of garlic EO against *S. littoralis* larvae while mitigating potential negative effects on plants (i.e., phytotoxicity), an insecticide nano-formulation was developed following the methodology described by Palermo et al. [19]. The developed formulation was characterized by a high amount of EO (i.e., 15%) and relatively low content (i.e., 5%) of surfactant (Tween 80) with an oil:surfactant ratio of 3:1. The preparation process involved mixing 15 g of garlic EO with 5g of Tween 80 using a magnetic stirrer (7000 RPM) for 30 min. Subsequently, distilled water (80 g) was slowly added to the mixture at 1 mL per minute. The resulting coarse emulsion was stirred for three hours (7000 RPM) and then sonicated (100 W for 90 s for 3 cycles) to enhance its stability and homogeneity. To avoid degradation of the garlic EO caused by the heat generated during sonication, the entire process was carried out using an ice bath. The raw garlic EO used in the experiments was purchased from Esperis s.p.a. (Milan, Italy) and belonged to the same batch (OL.ES. 4 20/2) as that used by Modafferi et al. [23], which provided complete analytical procedures and chemical characterization. A Dynamic Light Scattering (DLS) apparatus (Zetasizer Nano, Malvern, UK) was used to analyze the physical characteristics of the developed garlic EO-based nanoemulsion. The mean droplet size, the polydispersity index (PDI), and the droplet surface charge, indicated by the zeta potential (ζ) values, were assessed by diluting the nanoemulsion with double-distilled water (1:400 *v*:*v*).

### 2.3. Laboratory Bioassay on S. littoralis Larvae

The experiments were carried out at the laboratories of General and Applied Entomology of the Department of Agriculture of the University of Reggio Calabria (Italy) under controlled environmental conditions in growth chambers (25 ± 2 °C, 60 ± 10% RH, 14:10 h L:D). Seven different concentrations of garlic EO (0, 0.5, 1, 1.5, 2, 2.5, and 3%) were tested, and water was used as negative control. Based on preliminary observations, the tested concentrations were chosen to identify the dosage resulting in mortality levels ranging between 10% and 90% of the exposed larvae. This mortality range was selected to accomplish the Probit analysis requirement to adequately calculate the concentration of EO required to kill 50% (LC_50_) and 90% (LC_90_) of the exposed larvae. Chlorantraniliprole (Altacor^®^, DuPont Mississauga, Mississauga, ON, Canada) was used as a positive control. *Spodoptera littoralis* larvae were exposed to the maximum label dose for peppers (12 g/hL Altacor^®^, corresponding to 0.042 g/L of chlorantraniliprole).

The insecticidal activity of the developed formulation was assessed against the second instar larvae of *S. littoralis* using the leaf dip method. In detail, fresh bell pepper-leaf discs with a diameter of 5.2 cm were dipped into the different dilutions of the nano-formulation for 10 s and left to air dry for 45 min. After drying, each treated leaf disc was carefully placed in a sterile plastic arena (5.5 × 3.5 cm). Afterward, five larvae were released into each arena using a fine paintbrush. Once the larvae were placed inside the arena, the arenas were covered with a mesh to avoid any escape, allow ventilation, and prevent fumigation effect. For each concentration tested, including the controls (water and chlorantraniliprole), six replicates (arenas) were provided. Mortality was recorded daily for three days following the treatment. The larvae were considered dead if they did not display any movement upon stimulation with a fine paintbrush. In addition, the leaf surface eroded by larvae during the experiments was evaluated by ImageJ^®^ v.1.53 software (National Institutes of Health, Bethesda, MD, USA) and expressed as a percentage of eaten leaf disc.

### 2.4. Nontarget Effects on Plants

A specific trial was set up to evaluate the effects of the EO-based nano-formulation treatments on plants. This experiment was carried out at the agricultural research center Agrigeos s.r.l. (Acireale, Italy), in an experimental greenhouse. Fifteen days after plant emergence, these were transplanted into pots (two plants/pot) and fertilized using a nitrogen-based fertilizer.

Bioassays were carried out by spraying plants once until runoff, with the most effective concentrations tested against the second instar larvae of *S. littoralis* (i.e., 2, 2.5, and 3%). Similarly to the pest bioassays, water was used as negative control. Each treatment was replicated six times.

The number of healthy and damaged leaves and fruit, the severity of damage, and the plants’ height were recorded and counted for each pot; the amount of chlorophyll contained within the leaves, as measured by the SPAD-502Plus (Konica-Minolta, Osaka, Japan) optical sensor, was also registered. The effect of the developed formulation on plants was estimated using the phytotoxicity index (P_i_) proposed by Campolo et al. [14] and calculated as follows:

(1) $$P_{i}=\sum_{j=0}^{n}\left(\frac{{DL}_{j}}{TL}\times \frac{DC}{n-1}\right)$$

where *DL* is the number of damaged leaves for each damage severity class *j*, *TL* is the total number of leaves sprayed, *DC* is the damage severity class, and *n* is the number of damage-severity classes (0 = no phytotoxicity damage; 1 = 25% of damaged leaf surface; 2 = 50% of damaged leaf surface; 3 = 75% of damaged leaf surface; and 4 = 100% of damaged leaf surface). The calculated P_i_ ranges from 0 (no damage) to 1 (dead leaves). Plant damage was estimated daily for the first 7 days after treatment. After this period, damage was evaluated every 7 days. At the end of the experiments (i.e., 52 days), the plants were cut and weighed with and without fruit. Additionally, the radical apparatus was weighed by first removing the soil from the roots.

### 2.5. Data Analysis

Statistical analyses were carried out by using IBM SPSS 19 (Supplementary Material S1). Mortality rates, eroded leaf surfaces from *S. littoralis* bioassays, and data from phytotoxicity trials were subjected to ANOVA statistical procedure followed by HSD Tukey post hoc. All the data satisfied the ANOVA assumptions in terms of normality and homoscedasticity of variance (*p* > 0.05). Probit analysis was used to estimate LC_50_s and LC_90_s. Values were significantly different if their 95% fiducial limits did not overlap. This analysis provided insights into the potency and efficacy of the formulation. The obtained results were interpreted based on *p*-values (α = 0.05).

## 3. Results

### 3.1. Characterization of Garlic EO Nanoemulsion

**Figure 1:**
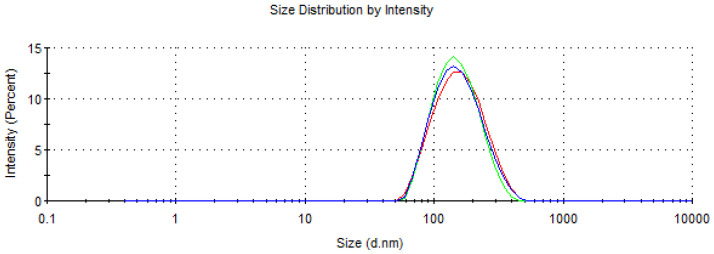
Size distribution of garlic EO-based formulation. Different colors represent different replicates.

**Figure 2:**
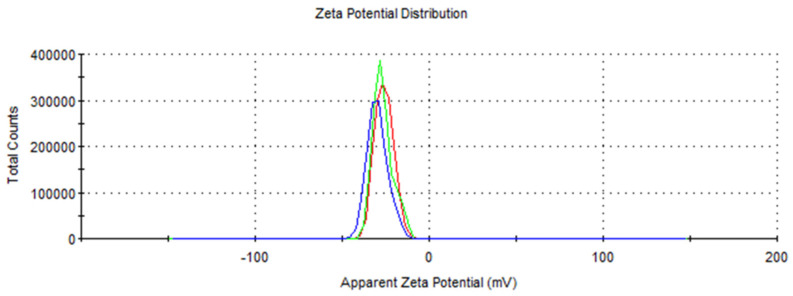
Zeta potential (ζ) of garlic EO-based formulation. Different colors represent different replicates.

The developed formulation was characterized by an average particle size of 141.0 ± 1.375 nm and a relatively low (0.146 ± 0.009) polydispersity index (PDI), suggesting a relatively narrow size distribution and excellent uniformity. In addition, the nanoemulsion exhibited a surface charge of −27.4 ± 1.91 mV, indicating the presence of a negative charge on the particle surfaces. During the experiments, no evidence of phase separation of the developed nano-insecticide was highlighted, confirming the stability of the nanoemulsion system (Figure 1 and Figure 2).

### 3.2. Laboratory Bioassays

The garlic EO-based nano-formulation exhibited high toxicity against second-instar larvae of *S. littoralis*. No mortality was recorded in the untreated control during the entire observation (72 h), whereas 100% mortality was recorded in the positive control.

The mortality induced by the developed formulation was significantly different among the application rates used (F = 86.70; df = 8; *p* < 0.01), whereas the time from the treatments did not affect the number of dead larvae (F = 1.623; df = 8; *p* = 0.21). The data related to mortality fitted the probit model (Table 1), and the results of the different LC values confirm that the efficacy of the developed formulation exerts its larvicidal action mainly during the first 24 h after treatment. Indeed, the LC values highlighted no statistical differences among the three different exposure times (i.e., fiducial limits did not overlap). The amounts of EO required to kill 50 and 90% of the exposed larvae after 24 h were 1.72 and 2.79%, respectively.

**Figure 3:**
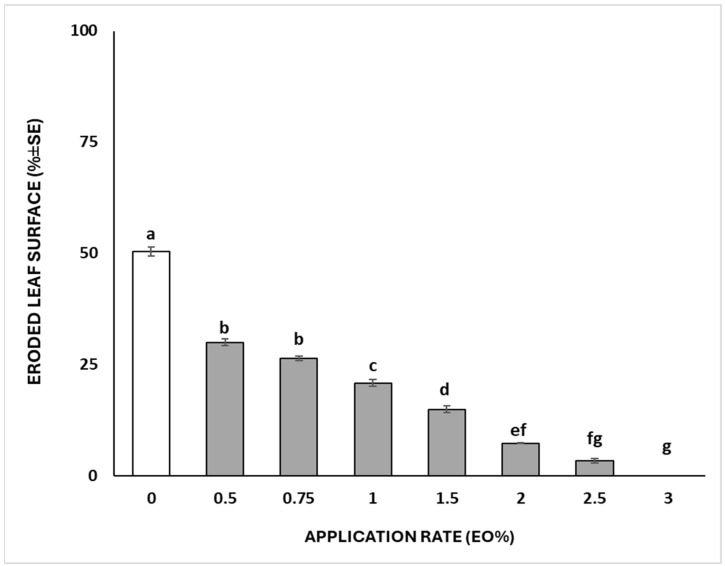
Percentage (mean ± SE) of disc leaf eroded by *S. littoralis* second instar larvae 72 h after the treatments. Different letters indicated significant differences among the groups according to Tukey’s HSD post hoc test at *p* ≤ 0.05.

**Figure 4:**
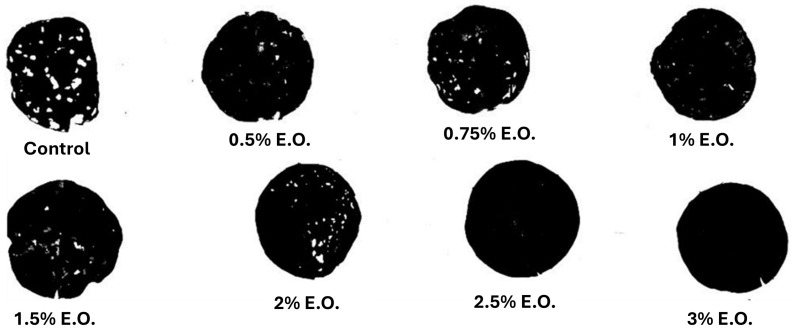
Example of eroded disc leaves at different doses of garlic EO processed with ImageJ v. 1.53 software.

The antifeedant effect of the garlic EO-based formulation is depicted in Figure 3 and Figure 4. The eroded leaf surface after the treatment followed a dose-dependent pattern (F = 336.51; df = 7; *p* < 0.001), where larvae were able to eat more than 50% of the untreated leaf area. Larvae treated with the maximum application rate (3% of EO) did not feed on leaves.

### 3.3. Effects of Garlic EO Nanoemulsion on Plants

**Figure 5:**
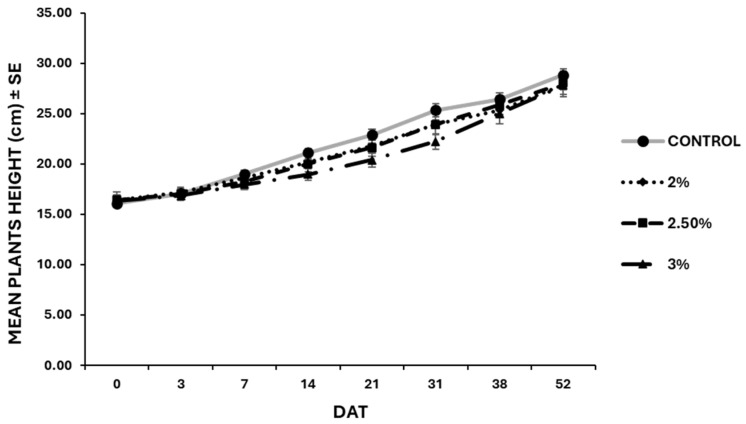
Plant height registered during the 52 days after treatment with garlic EO-based nano-formulation. No statistical differences (*p* > 0.05) were highlighted among the different treatments at the same time (*p* > 0.05). DAT = Days after treatment.

**Figure 6:**
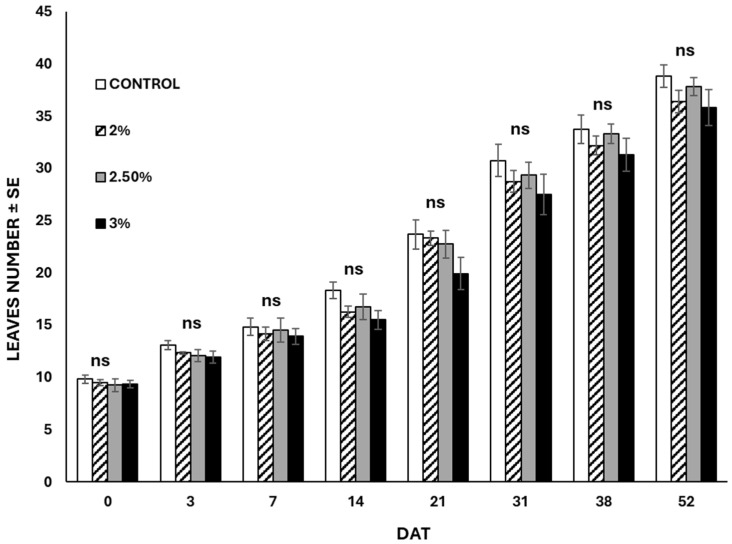
Number of leaves (mean ± SE) recorded in plants treated with garlic EO-based nanoemulsion at different application rates. No statistical differences (*p* > 0.05) were highlighted among the different treatments at the same observation time. DAT = Days after treatment; ns = not significant.

**Figure 7:**
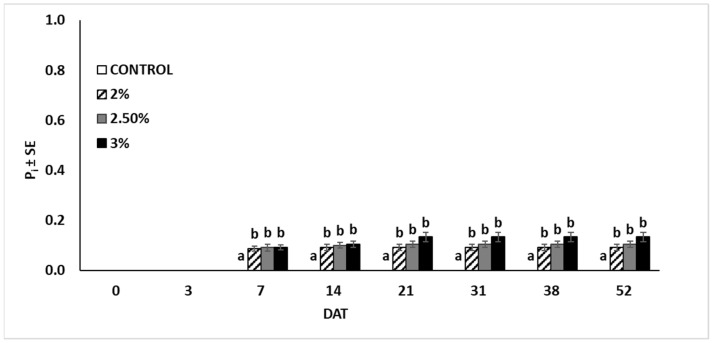
Phytotoxicity index (P_i_) on pepper leaves caused by garlic EO nanoemulsion appeared 7 days after treatment. The P_i_ increased up to 21 days after treatment and remained constant until the end of the trial. (Means ± SE) with different letters differ significantly according to Tukey’s HSD post hoc test at *p* ≤ 0.05. DAT = Days after treatment.

The results related to the plant growth exposed to the nano-formulation proved no statistical differences (*p* > 0.05 at all the sampling times) between the untreated control and the EO-treated plants (Figure 5). Indeed, all plants reached nearly the same height (about 30 cm). The recorded number of leaves per plant did not significantly vary across the different groups (*p* > 0.05 at all the sampling times) (Figure 6). Symptoms related to EO phytotoxicity appeared three days after treatment, and the number of symptomatic leaves continued to increase until 21 days after treatment, while the number of damaged leaves remained unchanged in subsequent assessments (Figure 7). In the treated plants, approximately four leaves per plant were damaged by the treatments, whereas in the untreated plants no leaves exhibited adverse symptoms throughout the experiment. The plants treated with the highest EO content (3%) displayed more severe symptoms (P_i_ = 0.13 ± 0.1) compared to those treated with the lowest EO dose (P_i_ = 0.09 ± 0.01).

**Figure 8:**
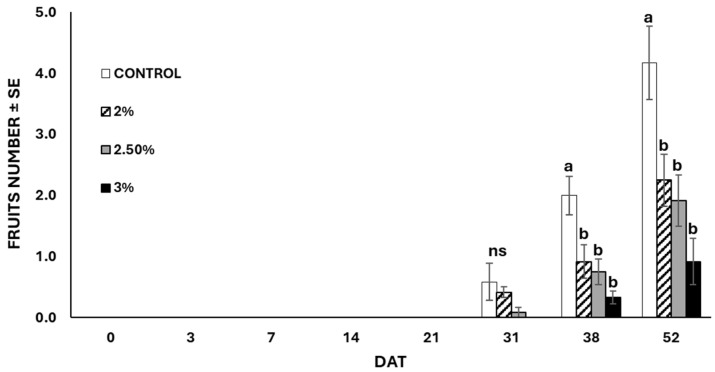
Exponential increase in the number of pepper fruits in plants treated and untreated with garlic EO nano-formulation. Fruits appear in both groups of plants 31 days after treatment. (Means ± SE) with different letters differ significantly according to Tukey’s HSD post hoc test at *p* ≤ 0.05. DAT = Days after treatment; ns = not significant.

**Figure 9:**
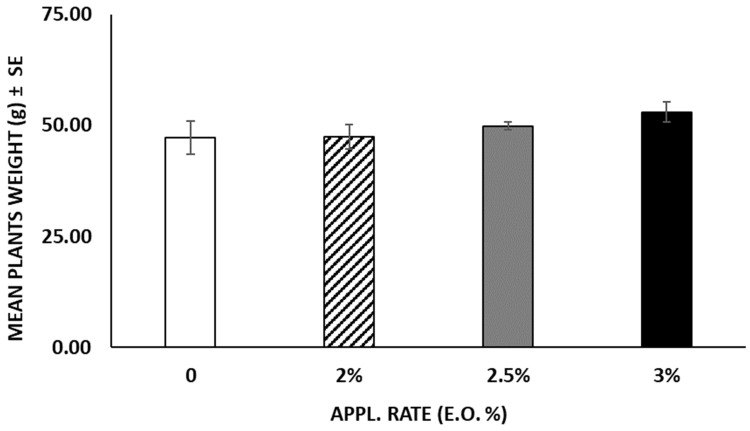
Mean plant weight (mean ± SE) was obtained at the end of the experiment after cutting and separating the roots into the soil. No statistical differences (*p* > 0.05) were highlighted among the different treatments. APPL. RATE = application rate.

**Figure 10:**
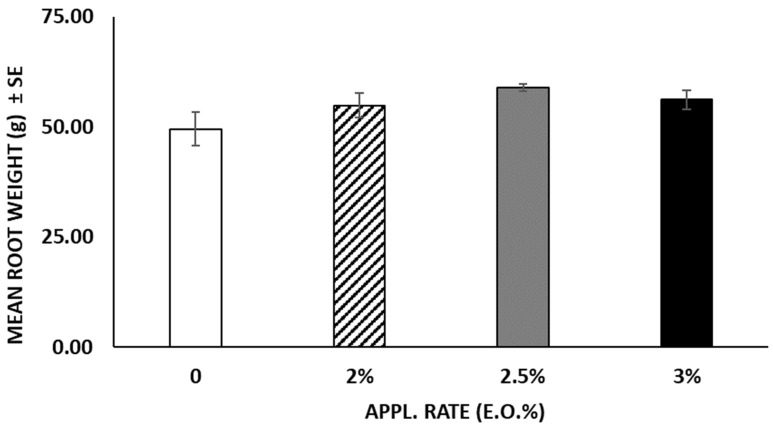
Mean root weight (mean ± SE) was obtained at the end of the experiment after cutting the plants. No statistical differences (*p* > 0.05) were highlighted among the different treatments. APPL. RATE = application rate.

**Figure 11:**
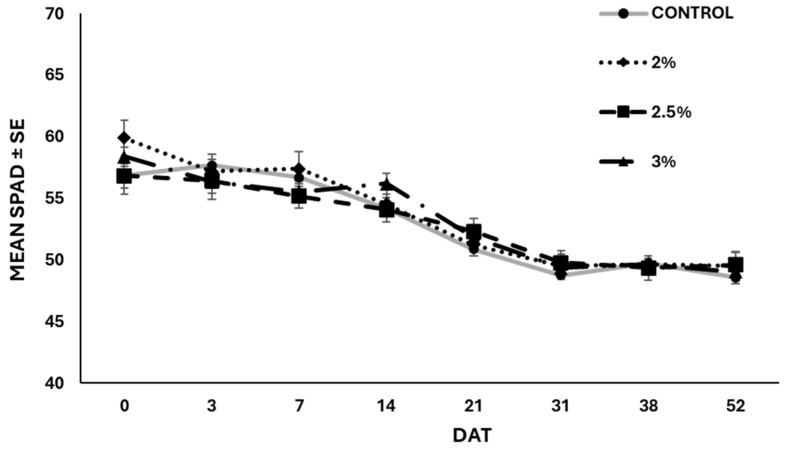
Decrease in chlorophyll inside the leaves (mean ± SE), measured by the SPAD optical sensor. No statistical differences (*p* > 0.05) were highlighted among the different treatments (ANOVA, Tuckey’s HSD post hoc test). DAT = Days after treatment.

In terms of fruits produced by the plants, untreated plants yielded an average of 4.2 ± 0.4 fruits per plant, whereas EO-treated plants produced significantly fewer fruits (F = 8.659; df = 3; *p* < 0.001) because of the different application rates (Figure 8). At the end of the experiment (52 days after the treatments), no statistical differences (F = 1.687; df = 3; *p* = 0.20) were observed among the weights registered in the different treatments. Plants treated with 3 and 2.5% of EO reached a weight of 52.98 g and 49.82 g, respectively, whereas untreated plants weighed 47.22 g (Figure 9). Likewise, the roots of untreated plants were lighter compared to those of the treated plants, despite no statistical differences (F = 2.290; df = 3; *p* > 0.05) observed between treated and untreated plants (Figure 10). The chlorophyll content measured using the SPAD instrument decreased over time in both the treated and untreated plants, although without significant differences (F = 0.869; df = 3; *p* = 0.459) (Figure 11).

## 4. Discussion

The main objective of this study was to develop an *A. sativum* EO-based nanoemulsion characterized by a high concentration of the active ingredient (15%) for the control of the key horticultural pest, *S. littoralis*. Subsequently, we evaluated the potential phytotoxic effects of garlic EO nano-formulation on bell pepper plants under greenhouse conditions.

The physical characteristics of the developed insecticidal formulation indicated a good quality both in terms of size and PDI, as well as in terms of stability. These parameters are essential for assessing the quality of the nanoemulsion and predicting its stability over time [24]. Nanoemulsions, possessing small droplet sizes (<200 nm), are considered favorable for their application against insect pests. Furthermore, the low PDI value suggests a highly homogeneous emulsion, which is less susceptible to the destabilization phenomenon known as Ostwald ripening [25]. The EO formulation proposed in this study had a mean particle size below 141 nm and a PDI near zero, confirming its good quality. Similar results were obtained by Ricupero et al. [26], who developed a nanoemulsion of garlic EO using the same self-emulsification process combined with sonication. Indeed, these authors obtained a slightly larger droplet size (176.23 ± 0.9 nm) and a less-homogeneous formulation (PDI = 0.18) compared to the one obtained during this research (PDI = 0.146). Since the methodology and procedure were the same, the differences between the two nanoemulsions, the present one and that produced by Ricupero et al. [26], can be attributable to the different batches of garlic EO used. On the other hand, Modafferi et al. [27], using a similar procedure, obtained particle size, PDI, and zeta potential comparable to those obtained in this study (size = 159 nm; PDI = 0.18; zeta potential = −21.9 mV). This evidence suggests that the EO composition is key in formulating EO-based nano-delivery systems.

Variability in the composition of EOs is common. It can depend on various factors, such as the geographical origin, the season of growth and harvesting, and the cultivar or variety used [28,29]. As an example, perillaldehyde was the main constituent of the oil of *ippia. javanica* var. *javanica* (Verbenaceae). At the same time, myrcenone (ipsdienone) was the main compound in the oils of *L. javanica* var. *whytei* [30]. In addition, the same authors highlighted the fact that while perillaldehyde, linalool, and carvone, components of the oil of *L. javanica* var. *javanica*, were toxic to adult *Sitophilus zeamais* (Motschulsky) (Coleoptera: Curculionidae), myrcenone, the other main component of the oil, was not; thus, plant variety can impact the EO insecticidal activity against target pests. The same authors also observed that EO yield and chemical composition varied significantly with harvest time during the season, for both varieties.

The physical properties of a nano-formulation can also be affected by the other ingredients and co-formulants used for its production. For example, a high relative amount of surfactant ensures the production of tiny droplets with an optimal size distribution (i.e., PDI tending towards zero). Nevertheless, the amount of surfactant should be reduced as much as possible when developing insecticides for crop protection, as suggested by Modafferi et al. [23], because of their phytotoxicity. In addition, the quality parameters (i.e., size, PDI, and particle surface charge) are strongly influenced by the approach used to prepare the nanoemulsion, as reported by Modafferi et al. [23], who used the same oil batch but had a different preparation process (i.e., high-pressure fluidization).

Regarding the insecticidal efficacy of the *A. sativum* EO-based nano-formulation, our findings suggest its potential for the control of *S. littoralis*. Indeed, almost 100% mortality of larvae exposed to the treatments was achieved at the two highest-tested concentrations. The efficacy of garlic EO against crop pests has already been assessed against other insect pest groups, such as Hemiptera [23], Lepidoptera [26,31], Isoptera [32], Coleoptera ([33,34]), Mallophaga [35], and Diptera [36], as well as against other arthropods, such as Acari [16]. On the other hand, few studies on garlic EO or extracts applied as insecticides against *S. littoralis*. Ali et al. [37] tested garlic EO aqueous solutions using Triton X-100 as an emulsifier for evaluating mortality and anti-feeding activity against *S. littoralis* fourth instar larvae. The authors highlighted the LC_50_ and LC_90_ values of 19.95% and 39.18% (*w*/*w*) of garlic EO, respectively, while the LC_50_ and LC_90_ values for lemon EO were higher (24.20% and 47.07%, respectively). Furthermore, garlic EO at a low concentration (LC_30_ = 16.30% of EO) revealed antifeedant activity with a reduced castor leaf consumption ranging from 62% to 74%, depending on the time post-treatment [37]. Our results showed significant antifeedant effects of the garlic EO nano-formulation, which varied depending on the application rates. However, at the maximum application rate (3% of EO), the exposed larvae did not feed, while at the lowest dose (0.5% of EO), only 30% of the disc leaves were consumed by larvae. Our results suggested that garlic EO nanoemulsion highly improved the bioactivity of crude EO, by reducing the amount of EO needed to kill or deter *S. littoralis* larvae. Thus, nano-formulation could have practical application in the field, while the amount required for pure EO could impair its use. In addition, EO-based nanoemulsion guarantees high stability and long persistence in the field, proving effective bioactivity and pest control for at least 72 h after plant treatment.

Apart from garlic EO, other EOs revealed promising insecticide and antifeedant activity against *S. littoralis* (reviewed in [38]). As an example, *Artemisia monosperma* (Asteraceae), *Callistemon viminals* (Myrtaceae), *Citrus aurantifolia* (Rutaceae)*,* and *Cupressus macrocarpa* (Cupressaceae) exhibited good insecticidal activities, affecting larval growth and feeding ability of fourth instar larvae, depending on the doses used in the trials [39]. From the literature, it has been highlighted that the insecticidal activity of EOs was tested exclusively under laboratory conditions against *S. littoralis,* and almost one-third of the research involved Lamiaceae EOs. Nevertheless, several biological activities were investigated (i.e., repellence and deterrence, larvicidal, ovicidal, and biochemical effects, and growth regulation), although most substances acted as antifeedants and contact insecticides [38]. Nevertheless, most of the articles reviewed by Jbilou et al. [38] tested EO bioactivity at less than 48 h only on third- and fourth-instar larvae. Here, we tested the mortality until 72 h, and we selected second-instar larvae, which is considered the best-targeted stage for effective pest control.

To the best of our knowledge, the negative effects of *A. sativum* EO used as a pesticide on bell pepper plants (i.e., phytotoxicity) have not been previously investigated. Nevertheless, a garlic EO-based nano-formulation had a negligible phytotoxic effect on tomato plants for up to 14 days after the insecticide treatment against *T. absoluta* [26]. In contrast, our results indicated plant damage beginning a few days after treatment. In pepper plants treated with the highest dose (3% of EO), leaf damage was observed in 8% of the plants just three days after treatment. This percentage increased to 13% by 21 days after treatment, resulting in approximately four damaged leaves per plant. Furthermore, significant differences were observed in the number of fruits produced among plants treated with different amounts of garlic nanoemulsion compared to untreated ones, suggesting a negative impact on fruit setting. These findings underscore the need for further investigations into the potential non-target effects of *A. sativum* EO on plant health and highlight the importance of careful consideration in pesticide application to minimize unintended harm to crops.

## 5. Conclusions

The quality of the garlic EO nanoemulsion was promising, and the evaluated physical parameters indicated good stability and potential efficacy against the target pest. Indeed, a good larvicidal activity of the EO formulation was recorded, suggesting its possible application in biological and/or integrated control programs to control *S. littoralis* larvae. Garlic EO-based nanoemulsion induced significant mortality in the target pest and, contextually, markedly reduced larval feeding activity in plants. Despite observing phytotoxicity symptoms in pepper plants when treated with the garlic EO nanoemulsion, these plants successfully completed their life cycle and produced fruit with slightly different outcomes than those of untreated plants. This aspect should be further investigated to evaluate interferences of garlic EO and its formulations for crop plants.

## Figures and Tables

**Table 1:** Acute toxicity of garlic EO nanoemulsion against the second instar larvae of *S. littoralis* 24, 48, and 72 h after the exposure. Values are expressed in percentage of EO and are considered significantly different if their 95% fiducial limits (i.e., values shown in parenthesis) do not overlap.

Time	LC ^a^_50_ (FL) ^b^	LC_90_ (FL)	χ^2 c^ (df ^d^)	*p* ^e^
24 h	1.72 (1.56–1.88) ^a^	2.79 (2.48–3.31) ^a^	33.58 (46)	0.91
48 h	1.57 (1.43–1.70) ^a^	2.39 (2.15–2.76) ^a^	41.35 (46)	0.67
72 h	1.57 (1.43–1.70) ^a^	2.39 (2.15–2.76) ^a^	41.35 (46)	0.67

^a^ Lethal Concentration; ^b^ Fiducial limits; ^c^ Pearson goodness of fit test; ^d^ Degree of freedom; ^e^ Probability value.

## Data Availability

The original contributions presented in the study are included in the article/Supplementary Materials, further inquiries can be directed to the corresponding author.

## Supplementary Materials

The following supporting information can be downloaded at: https://www.mdpi.com/article/10.3390/insects15070476/s1, Spreadsheet S1: Original data presented in the study.
